# Crystal Structure Formation of CH_3_NH_3_PbI_3-x_Cl_x_ Perovskite

**DOI:** 10.3390/ma9030123

**Published:** 2016-02-24

**Authors:** Shiqiang Luo, Walid A. Daoud

**Affiliations:** School of Energy and Environment, City University of Hong Kong, Tat Chee Avenue, Hong Kong, China; sqluo3-c@my.cityu.edu.hk

**Keywords:** solar cells, perovskites, X-ray diffraction, phase transitions

## Abstract

Inorganic-organic hydride perovskites bring the hope for fabricating low-cost and large-scale solar cells. At the beginning of the research, two open questions were raised: the hysteresis effect and the role of chloride. The presence of chloride significantly improves the crystallization and charge transfer property of the perovskite. However, though the long held debate over of the existence of chloride in the perovskite seems to have now come to a conclusion, no prior work has been carried out focusing on the role of chloride on the electronic performance and the crystallization of the perovskite. Furthermore, current reports on the crystal structure of the perovskite are rather confusing. This article analyzes the role of chloride in CH_3_NH_3_PbI_3-x_Cl_x_ on the crystal orientation and provides a new explanation about the (110)-oriented growth of CH_3_NH_3_PbI_3_ and CH_3_NH_3_PbI_3-x_Cl_x_.

## 1. Introduction

Since the first organic-inorganic halide perovskite solar cell was reported [1], perovskites have attracted growing interest and the power conversion efficiency (PCE) has reached 20.1% [2]. It is not very common that a photovoltaic device can experience such a rapid development. While the structure of the cells evolved from sensitized meso-structure to planar structure [3], both inorganic and organic materials can be applied as electron and hole transfer materials [4]. Furthermore, by tuning the composition of the perovskite, the band gap can be easily modified [5]. Given the numerous advantages of perovskite, a clear understanding of the crystal structure is crucial and the role of chloride in the formation of CH_3_NH_3_PbI_3-x_Cl_x_ (hereafter, we use MA short for CH_3_NH_3_) is one of the most pressing topics.

It has been reported that the presence of chloride in the perovskite improves the uniformity of its layer [6] and results in an increase of the carriers’ diffusion length from *ca.* 100 nm to over 1 μm [7]. However, the long held debate over of the existence of chloride in the perovskite seems to have now come to a conclusion. First, when synthesizing the perovskite by the one step method with precursor solution of MACl and PbI_2_ (1:1 molar ratio) in anhydrous N,N-dimethylformamide (DMF), the resulting crystal is not MAPbI_2_Cl but a mixture of MAPbI_3_ and MAPbCl_3_ [8]. This provides direct evidence that chloride (Cl^−^) cannot substitute iodine (I^−^) in the perovskite to form a stable crystal. Then, two contradictory results were then reported. X-ray photoelectron spectroscopy (XPS) showed that the molar ratio C:N:Pb:I:Cl of the perovskite is *ca.* 1:1:1:2:1, when prepared from a precursor of MAI:PbCl_2_ (molar ratio 3:1) [9]. On the other hand, energy dispersive X-ray (EDX) analysis showed that no Cl^−^ was present in the perovskite prepared from PbI_2_ + MAI + MACl [10]. Noting that the XPS was unable to determine the existence of MAPbI_2_Cl crystal and that EDX has its detecting limitation, more precise characterizations were needed. Later on, the simultaneous Fourier transform infrared spectroscopy analysis of the expelled gas during the decomposition of MAPbI_3-x_Cl_x_ showed the presence of Cl^−^, angle-resolved XPS [11] and X-ray fluorescence spectroscopy (XFS) [12] not only confirmed the existence of Cl^−^, but also showed that Cl^−^ was located at the interface between the perovskite and the electron transport TiO_2_ layer, and not in the perovskite structure [11,12]. Moreover, scanning transmission microscopy-energy dispersive spectroscopy (STEM-EDS) detected no trace of Cl^−^ in the perovskite. Even though there is a strong Cl^−^ signal, no N was observed indicting the presence of only PbCl_2_ [13]. Thus, Cl^−^ only appears at the interface between MAPbI_3_ and the anode. Two more reports have further confirmed this conclusion. XPS analysis showed only weak Cl^−^ signal after etching the surface of MAPbI_3-x_Cl_x_ by a 50 nm thickness [14]. Hard X-ray photoelectron spectroscopy and fluorescence yield X-ray absorption spectroscopy showed no Cl^−^ at the surface of MAPbI_3-x_Cl_x_ with higher average concentration of Cl throughout the perovskite layer at the deep beneath [15]. Here, we refer to MAPbI_3-x_Cl_x_ as MAPbI_3_ that is prepared using chloride-containing precursors. However, as the condition for depositing MAPbI_3-x_Cl_x_ differs, Cl^−^ may still remain in the resulting perovskite layer. For instance, X-ray absorption near edge structure (XANES) results showed that x = 0.05 ± 0.03 Cl atoms per formula unit remain in the films after annealing at 95 °C for 120 min [16]. The results from photothermal induced resonance (PTIR) showed that the MAPbI_3-x_Cl_x_ film consists of a mixture of Cl-rich (x_local_ < 0.3) and Cl-poor phases after a mild annealing (60 °C, 60 min) and homogeneous Cl-poorer (x_local_ < 0.06) phase upon further annealing (110 °C) [17].

In addition, first-principles calculation results provide some good explanation. For the crystal structure, Cl^−^ concentration was found below 3%–4% [8] and if the Cl^−^ ions enter the crystal structure, they preferentially occupy the apical positions in the PbI_4_X_2_ octahedra [18]. For the electronic property, while the molecular orientations of CH_3_NH_3_^+^ result in three times larger photocurrent response than the ferroelectric photovoltaic BiFeO_3_, Cl^−^ substitution at the equatorial site induces a larger response than does substitution at the apical site [19]. Results also showed that, using Cl^−^ precursor can avoid forming the PbI defects [20]. Introducing Cl^−^ would reduce the lattice constant which can inhibit the formation of interstitial defects [21]. As excitons may be screened by collective orientational motion of the organic cations, Cl^−^ might hinder this motion and results in better transport properties [22]. Little difference of electronic properties was represent among orthorhombic, tetragonal and cubic phases of MAPbI_3_ [23], however, the valance-band-maximum and conduction-band-minimum states can be mainly derived from iodine ions at some unique positions, Cl^−^ substitution can strengthen the unique position of the ions and result in more localized charge density [24]. Thus, lower carrier recombination rate and enhanced carrier transport ensued. For the interface, the (001) and (110) surfaces tend to favor hole injection to 2,2′,7,7′-tetrakis(*N,N*-di-*p*-methoxyphenylamine)-9,9′-spirobifluorene (Spiro-MeOTAD), while the (100) surface facilitates electron transfer to [6,6]-phenyl C_61_-butyric acid methyl ester (PCBM) [25]. A better structural matching between adjacent rows of perovskite surface halides and TiO_2_ under coordinated titanium may be the reason for the (110)-oriented growth of MAPbI_3-x_Cl_x_ and MAPbI_3_ [26]. Interfacial Cl^−^ may thus further stabilize the (110) surface and modify the interface electronic structure between MAPbI_3_ and TiO_2_ [26].

Despite the absence of Cl^−^ in the perovskite, it still played an important role in the crystallization process. For instance, the morphology of MAPbI_3-x_Cl_x_ was compared with MAPbI_3_ [27] and a model in which the Cl^−^ rich phase modifies the morphologies of perovskite was proposed and fit well with the results from scanning electron microscopy (SEM) [27]. In addition, the transmission electron microscopy (TEM) of freeze-dried perovskite MAPbI_3−x_Cl_x_ precursor solution showed the presence of PbCl_2_ nanoparticles [28] and this is in agreement with the dynamic light scattering (DLS) investigations of MAPbI_3−x_Cl_x_ precursor solution [29]. Thus, references [28,29] further proved the model of the heterogeneous nucleation by PbCl_2_ nanoparticles proposed in reference [27]. However, the formation mechanism of the crystal structure remains undermined and this will be discussed in the following parts of this article.

## 2. Methods for Fabricating MAPbI_3-x_Cl_x_

In Section 3, we discuss the crystal structure of MAPbI_3-x_Cl_x_ according to the deposition method. As the fabrication methods were discussed in detail in reference [30], here we add a brief introduction about the preparation methods of MAPbI_3-x_Cl_x_. For the one-step deposition method, MAI:PbI_2_/PbCl_2_ (molar ratio 1:1 or 3:1) [31,32] were dissolved in γ-butyrolactone (GBL) or DMF, spin-coated on the substrates and annealed to form perovskite. Different annealing conditions result in different morphology of the MAPbI_3-x_Cl_x_ layer. While a rapid thermal annealing at 130 °C resulted in micron-sized perovskite grains [33], two-step annealing, such as 90 °C for 30 min then at 100 °C for 2 min [34] or 60 °C then ramping to 90 °C [35], resulted in optimal PCE on poly(3,4-ethylenedioxythiophene) poly(styrene-sulfonate) (PEDOT:PSS) substrates. A full coverage of perovskite can be achieved by rapid cooling after annealing [36]. To increase the solubility of Cl^−^, 1,8-diiodooctane [37] or other alkyl halide additives [38] or dimethyl sulfoxide [9] can be employed. Adding poly-(vinylpyrrolidone) (PVP) can also improve the surface coverage of perovskite [39]. It is interesting to note that, for MAPbI_3−x_Cl_x_, a simple annealing step is enough to form a good coverage [6,40], but for MAPbI_3_, a special step, such as multi-deposition [41], adding N-cyclohexyl-2-pyrrolidone (CHP) [42], fast deposition [43,44,45], or air flow during spin coating [46,47], is needed.

The sequential deposition method was mainly applied for MAPbI_3_ perovskite. In a typical synthesis, the solution of PbI_2_ in DMF was spun on a substrate as the first step then the substrate was dipped in a solution of MAI in 2-propanol (IPA) to form MAPbI_3_ crystals as the second step [48]. For the inclusion of chloride, in the first step the PbCl_2_ can be mixed with PbI_2_ in DMF or dimethyl sulfoxide (DMSO) [49,50,51,52], and/or the second step MACl can be added [53,54,55]. For vapor based deposition methods, the MAPbI_3-x_Cl_x_ can be formed by co-evaporating MAI and PbCl_2_ onto the substrates [56,57] or by reacting PbCl_2_ on substrates with MAI vapor [58,59].

## 3. The Crystal Structure Form and Formation

### 3.1. Crystal Structure of MAPbI_3_ Layer

**Figure 1:**
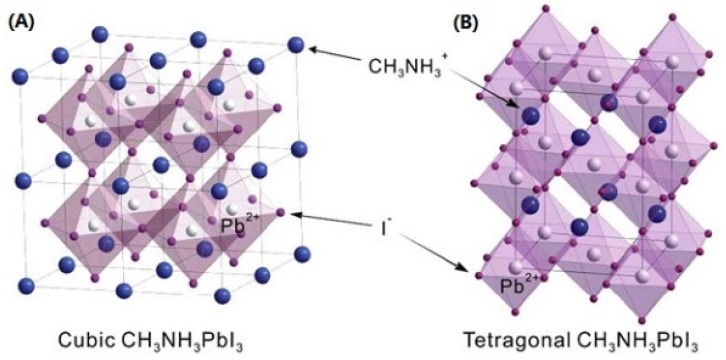

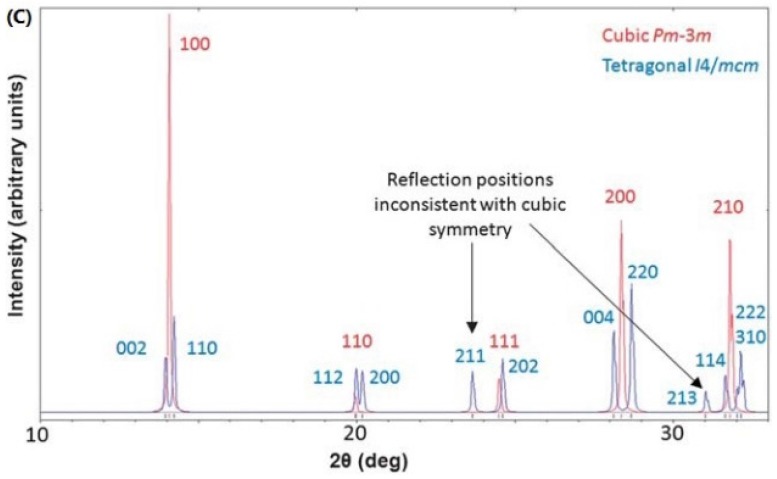
(**A**) Atomic models of MAPbI_3_ with cubic phase; and (**B**) tetragonal phase; (**C**) the calculated XRD patterns for MAPbI_3_ in both phases. (**A**) and (**B**) are reprinted from reference [64], Copyright © IOP Publishing. Reproduced with permission. All rights reserved; (**C**) is reprinted from reference [65], Copyright © 2013, Royal Society of Chemistry.

The parameters and transitions of phases of bulk MAPbI_3_ were included in references [60,61]. Here, we focus on the tetragonal and cubic phases [62]. In fact, there are no critical differences between the two phases, except a slight rotation of PbI_6_ octahedra along the c-axis. The atomic structures of MAPbI_3_ of the two phases are shown in Figure 1A,B. Thus, the tetragonal phase can be treated as a pseudocubic phase with a* = a/√2, c* = c/2 [63]. Below 54 °C, the cubic phase of MAPbI_3_ can be transformed into the tetragonal phase [60], and the opposite transition occurs by annealing at 100 °C for 15 min [41]. In Figure 1C, the X-ray diffraction (XRD) patterns of the two phases are shown. After transformation to the tetragonal phase, the (100) and (200) peaks of cubic MAPbI_3_ split, also new (211) and (213) peaks show up. Here, we use the peak splitting as indictor for phase transformation. Analysis of the MAPbI_3-x_Cl_x_ usually shows the cubic phase of MAPbI_3_, however, with a much more preference along (100) and (200). This will be discussed in the Section 3.2 and Section 3.3.

Another phase which should be noted is the amorphous phase. Pair distribution function analysis of X-ray scattering showed that after annealing at 100 °C for 30 min, the MAPbI_3_ in meso-porous TiO_2_ has about 30 atom% in medium range crystalline order and the other 70 atom% in a disordered state with a coherence length of 1.4 nm [66]. The poor crystallization of the MAPbI_3_ in meso-porous TiO_2_ was studied by high-resolution TEM [67]. Quartz crystal microbalance measurements suggest that during the sequential method only half of PbI_2_ is converted to MAPbI_3_ instantly, while the other half is involved in reversible transformation with MAPbI_3_. Additionally, the amorphous character with a very small average crystallite size may be present after the transformation as previously discussed [68]. The amorphous phase may also present during the initially deposited MAPbI_3-x_Cl_x_, as indicated by the envelope in some XRD spectra. In reference [69], the amorphous phase MA_5_PbCl_4_I_3_ was also mentioned. Moreover, both XRD and photoluminescence studies of MAPbI_2_Cl (2MAPbI_3_+MAPbCl_3_) indicate the existence of the amorphous phase [70].

### 3.2. Converting Lead Halides to Perovskite

In the sequential deposition method, PbI_2_ or/and PbCl_2_ were first dissolved in a solvent. As PbI_2_ crystal has a layered structure, DMF can intercalate into the PbI_2_ interlayer space and screen PbI_2_ via Pb-O bonding [71,72,73]. When DMF is intercalated, the XRD peak of the PbI_2_ (001) plane red shifts from 14.8° to 7.94° [72,73]. The red-shift of this XRD peak to 9.17° also indicates the intercalation of DMSO [43]. While PbCl_2_ doesn’t possess a similar layered structure as PbI_2_, its solubility is poor where PbCl_2_ nanoparticles may only suspend in the solvent [28]. However, depositing a mixture of PbI_2_ and PbCl_2_ on the substrates result in a new PbICl phase [74], whose crystal structure is similar to PbCl_2_ [75].

**Figure 2:**
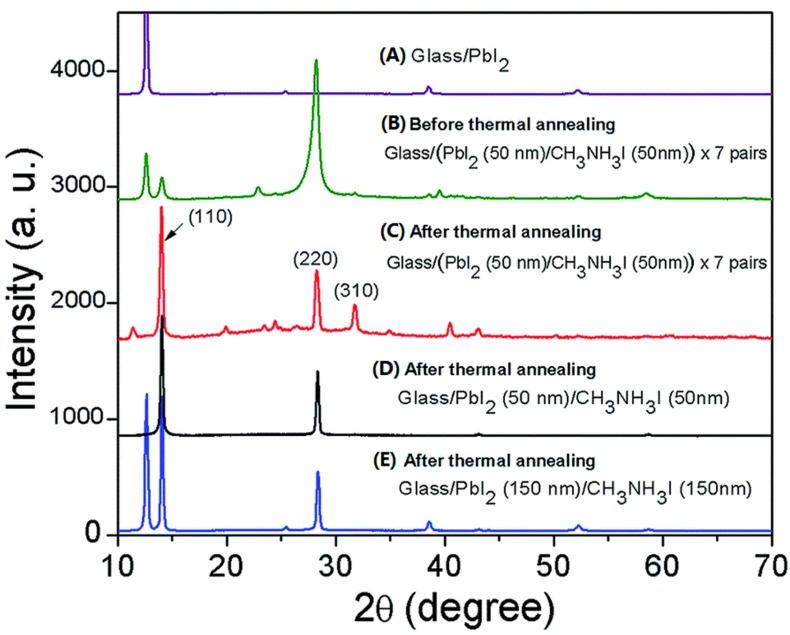
XRD patterns of glass substrates with vapor deposition of (**A**) a layer of PbI_2_; and (**B**) a layer of PbI_2_ followed by a layer of MAI, repeating this step 7 times; XRD pattern of the (**C**) annealed 7-time deposited PbI_2_/MAI layer; (**D**) 1-time deposited PbI_2_ (50 nm thickness)/MAI (50 nm thickness) layer; and (**E**) 1-time deposited PbI_2_ (150 nm thickness)/MAI (150 nm thickness) layer. Reprinted from reference [77], Copyright © 2015, Royal Society of Chemistry.

**Figure 3:**
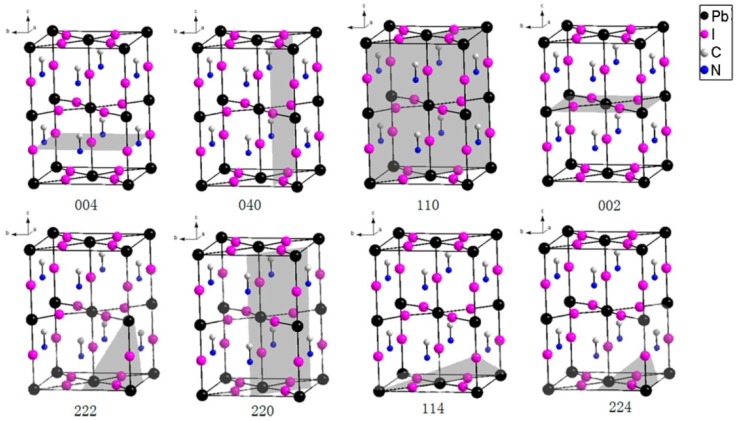
Crystallographic (lattice) planes (in gray) of tetragonal MAPbI3. Reprinted from reference [78], Copyright © 2015, American Chemical Society.

At the beginning of the reaction of PbI_2_ and MAI, a predominant peak at (220) appeared (as shown in Figure 2B). In other words, the MAPbI_3_ preferentially grows along (220) plane at first. The annealing process increases the long range crystalline order and results in the predominant (110) peak instead. Noting the (220) is only a short range of (110), thus, another possible reason for the (110)-oriented growth of MAPbI_3-x_Cl_x_ and MAPbI_3_ may be because layered crystal structure of PbI_2_ (growth along (001) planes of PbI_2_ like the liquid catalyst cluster model mentioned in reference [76]). The lattice planes of tetragonal MAPbI_3_ are showed in Figure 3.

For PbCl_2_, Cl^−^ was detached from PbCl_2_ when the PbCl_2_ was evaporated on the MAI substrate [79] and all the atoms of lead halide were dissociated during the crystal formation of the perovskite [80]. Thus, except the speed and the way of breaking the lead halide, the following steps should be similar with the one step method (Section 3.3) for converting PbI_2_ or PbCl_2_ with MAI to the perovskite. However, the situation in the presence of MACl may be different. As less energy is needed for MACl than MAI to undergo phase transition from solid to gas [69], it may be easier for MACl than MAI to diffuse into the PbI_2_ and cause the crystallization of perovskite [81]. However, as Cl^−^ cannot be incorporated into MAPbI_3_ crystal structure, the MAI and MACl may compete with each other to determine the result crystal, because only MAPbI_3_ or MAPbCl_3_ was formed when PbI2 was soaked in 80 mM MAI + 40 mM MACl or in 40 mM MAI + 80 mM MACl, respectively [80]. Thus, the incorporation of some amount of MACl managed to modify the morphology of the perovskite and resulted in better performance of the solar cells.

### 3.3. One Step Deposition of MAPbI_3-x_Cl_x_

The better crystallization of MAPbI_3-x_Cl_x_ along (110) and (220) plane of the tetragonal phase or (100) and (200) planes of the cubic phase may be due to the lowered cubic-tetragonal phase transition temperature of MAPbI_3−x_Cl_x_ after the incorporation of Cl^−^ [82]. A clear cubic-tetragonal phase transition temperature of MAPbI_3_ was detected by differential scanning calorimeter (DSC) analysis [65], however no such phase transition was observed for MAPbI_3−x_Cl_x_ [83]. To explain the absence of the phase transition for MAPbI_3−x_Cl_x_, we first study the crystallization process of MAPbI_3-x_Cl_x_ by one step deposition method.

**Figure 4:**
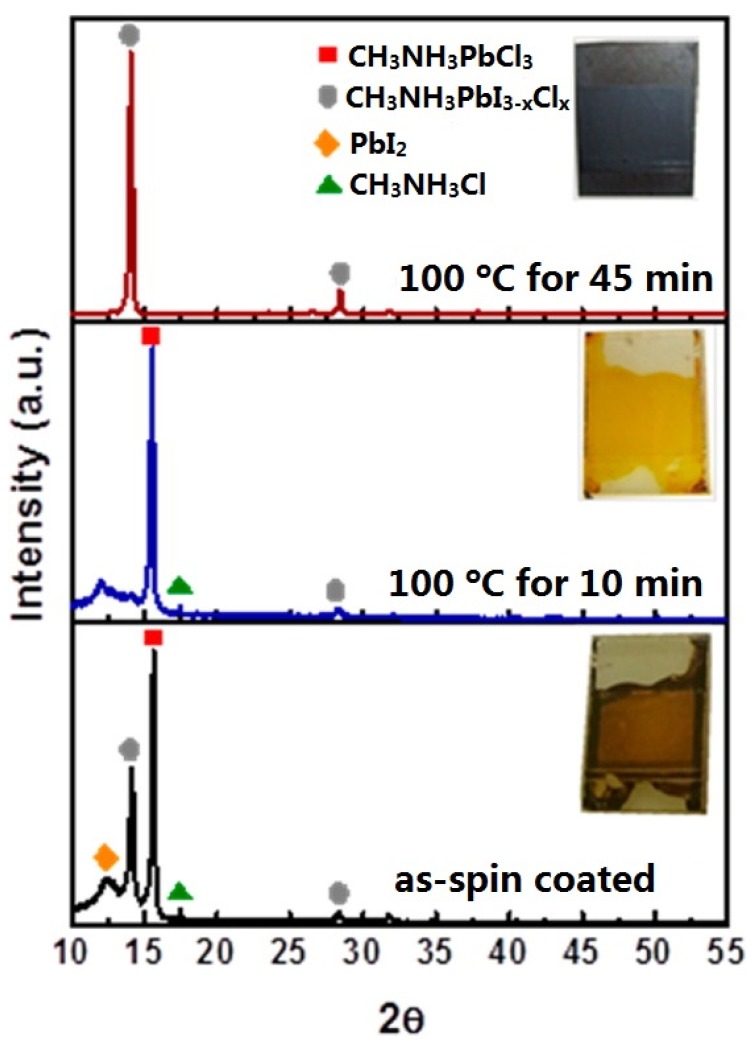
XRD patterns and optical images (insets) of MAPbI_3-x_Cl_x_ film during annealing. Reprinted from reference [84], Copyright © 2015, American Chemical Society.

Detail information about crystal formation process of MAPbI_3_ is summarized in reference [85]. For MAPbI_3-x_Cl_x_, the transformation from the intermediate phase to the perovskite is determined as 80 °C by *in situ* grazing incidence wide-angle X-ray scattering (GIWAXS) [86]. Figure 4 presents a clearer picture of the crystal formation of MAPbI_3-x_Cl_x_. The 15.7° and 31.5° peaks are associated with the (100) and (200) diffraction peaks of MAPbCl_3_ [82]. These peaks were also observed in references [27,87,88]. In Figure 4, it is interesting to note that MAPbI3 was formed first for the as-spin coated film but converted to MAPbCl_3_ after annealing at 100 °C for 10 min, and then MAPbCl_3_ was converted back to MAPbI_3_ after 45 min of annealing [84]. Further annealing would result in the decomposition of MAPbI_3_ to PbI_2_, but this occurred after conversion to the intermediate phase to MAPbI_3_ [89]. Because MAPbCl_3_ is in a cubic phase, we suppose that MAPbCl_3_ may cause a template effect for the cubic MAPbI_3_ phase.

In addition, the MAI:PbI_2_ (molar ratio 3:1) precursor solution on compact TiO_2_ can also form MAPbI_3_ with a predominant (110) plane, but the annealing temperature need to be above 150 °C [84,88,90]. The different sublimation temperature of MAI and MACl and the evidence of residue MAI or MACl in the resulting perovskite may explain the higher annealing temperature needed for MAPbI_3_ [84,91].

**Figure 5:**
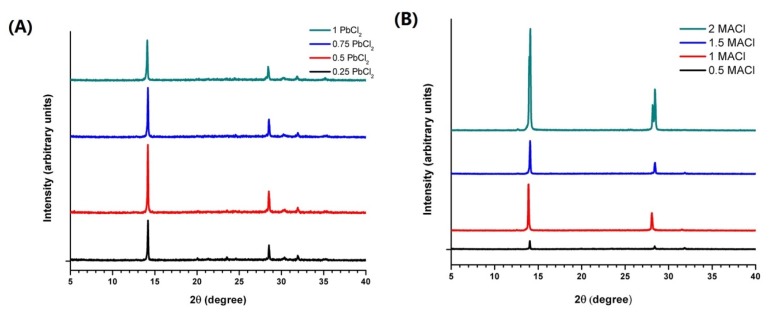
XRD patterns of MAPbI_3-x_Cl_x_ prepared from (**A**) precursor solution xPbCl_2_+yPbI_2_+zMAI (x = 0.25, 0.5, 0.75 and 1; y = 1 − x; z = 3 × x + y) in DMF; and (**B**) precursor solution 1PbI_2_+1MAI+xMACl (x = 0.5, 1, 1.5 and 2). Reprinted from reference [27], Copyright © 2014, American Chemical Society.

The XRD patterns of the resulting MAPbI_3-x_Cl_x_ prepared from different chloride-containing precursors are summarized in Figure 5. All the patterns showed predominant crystallization along the (110) and (220) planes. Interestingly, the (220) peak split at a high MACl (x = 2) concentration in Figure 5B [27]. This split was also observed in reference [29]. In the sequential deposition method, the (110) and (220) crystallization preference may be due to an *in situ* transformation process [92] of PbI_2_ to MAPbI_3_, as discussed in Section 3.2. However, the PbI_6_ octahedra are more likely to be fully dissociated in the one step precursor solution. [29,93,94,95] As MACl does not fit in the MAPbI_3_ structure, it could be possible that MACl may be expelled along the (110) planes of the MAPbI_3_ and that is why the MAPbI_3-x_Cl_x_ always showed the (110) and (220) orientation preference. This assumption can be proved by (220) peak split in Figure 5B, as excess of MACl breaks down the crystal range along (110) planes resulting in peak split. However, MAI can fit in the MAPbI_3_ structure, (110)-oriented growth is just the result of cubic phase in high temperature (150 °C in refereneces [84,88,90]). Surprisingly, a main XRD peak of (310) was observed for the one step deposition prepared MAPbI_3_ [96]. The main peak of (310), which is distinct from the (110) peak, may have resulted from the fact that the MAI was added into the precursor solution after the PbI_2_ was completely dissolved instead of both MAI and PbI_2_ being present at the same time [96], or the fact that the (310) plane of MAPbI_3_ may match the crystal structure of the substrate better. Then the magnitude of the (110) peak of MAPbI_3-x_Cl_x_ and the (310) peak of MAPbI_3_ further increases after 5 weeks [96]. Thus, we believe that the annealing process may only reinforce the crystallization preference as it is initially formed and the effects of substrate also contribute to the crystal structure formation of the perovskite in some cases. Returning to Figure 5, if excess of MACl breaks down the growth along the (110) plane, we believe MACl can also break down the crystalline order range. Since a large amount of MAPbI_3_ existed in the amorphous phase form, the cubic phase of MAPbI_3_ may be more favorable in short crystalline order range than the tetragonal phase.

There are other influences associated with Cl^−^. Increasing the temperature during the soaking of the PbI_2_ substrate in MAI + MACl IPA solution can improve the (110) orientation of MAPbI_3-x_Cl_x_ where the high temperature facilitates the expelling of MACl [97]. Annealing the MACl:PbI_2_ (3:1) precursor on compact TiO_2_ at 60 °C for 10 min followed by 100 °C for 20 min instead of gradually heating from 25 to 100 °C for 45 min resulted in the (200) crystal plane of MAPbI_3-x_Cl_x_ being vertically aligned on the substrate [98]. The tetragonal phase MAPbI_3−x_Cl_x_ was occasionally found on compact TiO_2_ substrate [53], while the cubic phase always occurred in meso-porous substrate, where the trapped MACl in meso-porous structure [8] helps the formation of cubic phase. While the size of MAPbI_3_ crystal grains are smaller but the degree of crystallinity improves in the presence of MACl [27,54], the sequential deposited MAPbI_3-x_Cl_x_ results in (001) elongated crystals [13].

## 4. Conclusions

In this article, the location of Cl^−^ and its influence on the crystal morphology of MAPbI_3-x_Cl_x_ is summarized, where the deposition methods (one step deposition, sequential deposition and vapor based deposition) for MAPbI_3-x_Cl_x_ are reviewed. Furthermore, the cubic and tetragonal phases of MAPbI_3_ are elucidated and the crystallization process of MAPbI_3-x_Cl_x_ is also summarized. Detailed information about the crystal structure with variable deposition parameters is also discussed. Though a recent report showed that Cl^−^ mainly improves the carrier transport at the perovskite/Spiro-MeOTAD and perovskite/TiO_2_ interfaces, rather than within the perovskite crystals, the authors of reference [99] more recently spatially resolved photoluminescence decay results showed less recombination in the high chlorine concentration region [100]. Thus, the effect of high concentration of Cl^−^ on the morphologies and electronic properties of the perovskite can still not be ignored. Additionally, whether Cl^−^ is predominantly present as a substituent for I^−^, as an interstitial, or at the surface of the crystal, remains unclear [101] and this is worth further investigation.
